# 1164. Risk of corneal transplant rejection and vaccination: a case-control study from a large, integrated health care system in Southern California

**DOI:** 10.1093/ofid/ofad500.1004

**Published:** 2023-11-27

**Authors:** Jennifer H Ku, Afshan Nanji, Julia Tubert, Cynthia Joe, Yi Luo, Kevin L Winthrop, Divya Srikumaran, Amanda E Brunton, Ana Florea, Frederick Fraunfelder, Tsai Yu Tseng, Hung Fu Tseng

**Affiliations:** Kaiser Permanente Southern California, Pasadena, California; Casey Eye Institute, Oregon Health & Science University, Portland, Oregon; Kaiser Permanente Southern California, Pasadena, California; Kaiser Permanente, Pasadena, California; Kaiser Permanente Southern California, Pasadena, California; OHSU-PSU School of Public Health, Portland, Oregon; Wilmer Eye Institute, Baltimore, Maryland; COPD Foundation, Miami, Florida; Kaiser Permanente Southern California, Pasadena, California; Oregon Health & Science University, Portland, Oregon; Kaiser Permanente Southern California, Pasadena, California; Kaiser Permanente Southern California, Pasadena, California

## Abstract

**Background:**

Vaccination is recommended for prevention of infectious diseases. For individuals with a history of corneal transplantation, however, there are concerns that vaccination may trigger allograft rejection through elevated immune activity. The association between vaccination and graft rejection remains unclear.

**Methods:**

We conducted a nested case-control study to evaluate the association between graft rejection and vaccination in corneal transplant recipients at Kaiser Permanente Southern California (KPSC) between January 2008 and August 2022. We identified all KPSC members who received a corneal transplant during the study period, with no history of prior transplant or rejection. Cases were those who experienced a graft rejection during the study period and eligible controls were those who had not experienced a rejection at the time of a given case. Controls were randomly selected via risk-set sampling and matched in a 3:1 ratio to cases on age, sex, and transplant date (±12 weeks). Index date was the date of rejection for cases. For controls, index date was determined by adding the number of days between transplant and rejection of the matched case to the control’s transplant date. We performed multivariable conditional logistic regression to compare the odds of vaccination (all types) in the 12 weeks prior to the index date in cases and controls, while adjusting for potential confounders.

**Results:**

Our study included 601 corneal transplant recipients with rejection (cases), and 1,803 matched controls. The overall cohort was 48% female and 47% white, with a mean age of 66 years (s.d. 17) (**Table 1**). A total of 136 (23%) cases and 373 (21%) controls received vaccination within 12 weeks of the index date (adjusted odds ratio 1.17, 95% CI: 0.91, 1.50) (**Table 2**).

**Table 1.**
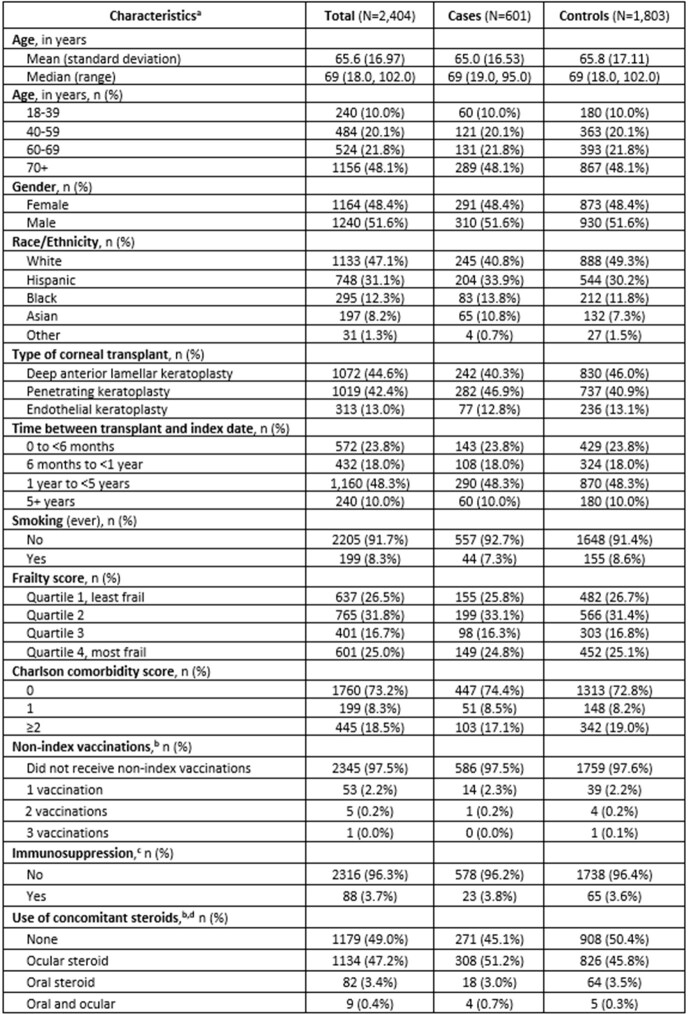
Characteristics of corneal transplant recipients who experienced graft rejection (cases) and who did not experience graft rejection (controls), Kaiser Permanente Southern California, 01/01/2008 - 08/31/2022. a Defined in the 12 months prior to index date unless otherwise indicated b Defined in the 12 weeks prior to index date c HIV/AIDS, leukemia, lymphoma, congenital immunodeficiencies, asplenia/hypospenia, or organ transplant at any time prior to index date, or immunosuppressant medication used during 12 weeks prior to index date d Includes dexamethasone, fluorometholone, loteprednol, difluprednate, prednisolone, methylprednisolone, and prednisone.

**Table 2.**
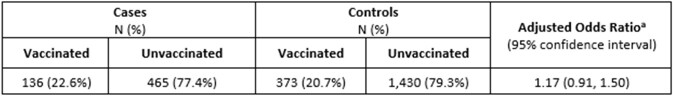
Vaccination among corneal transplant recipients who experienced graft rejection (cases) and who did not experience graft rejection (controls), Kaiser Permanente Southern California, 01/01/2008 - 08/31/2022. a Adjusted for race/ethnicity, Charlson comorbidity score, immunocompromised status, corneal transplant type, oral/ocular steroids received 12 weeks prior to index date (yes/no), non-index vaccinations received 12 weeks prior to index date (yes/no), and medical center and area.

**Conclusion:**

We did not find evidence of an elevated risk of graft rejection associated with vaccination given in the 12 weeks prior to the index date. Although our study is the largest study examining the association between corneal transplant rejection and vaccination to our knowledge, this study may be underpowered to detect a minimal increase in risk. Our findings provide support for completion of recommended vaccinations for patients who are planning or have received a corneal transplant.

**Disclosures:**

**Jennifer H. Ku, PhD MPH**, GlaxoSmithKline: Grant/Research Support|Moderna: Grant/Research Support **Julia Tubert, MPH**, Moderna: Grant/Research Support|Pfizer: Grant/Research Support **Yi Luo, PhD**, GlaxoSmithKline: Grant/Research Support|Moderna: Grant/Research Support|Pfizer: Grant/Research Support **Kevin L. Winthrop, MD, MPH**, AN2: Advisor/Consultant|AN2: Grant/Research Support|Insmed: Advisor/Consultant|Insmed: Grant/Research Support|Insmed: This study was funded by Insmed Inc.|Paratek: Advisor/Consultant|Paratek: Grant/Research Support|Red Hill Biopharma: Advisor/Consultant|Red Hill Biopharma: Board Member|Red Hill Biopharma: Grant/Research Support|Renovion: Advisor/Consultant|Renovion: Grant/Research Support|Spero: Advisor/Consultant|Spero: Grant/Research Support **Divya Srikumaran, MD**, Alcon: Advisor/Consultant|Claris Biotherpeautics: Grant/Research Support **Ana Florea, PhD MPH**, Gilead: Grant/Research Support|GlaxoSmithKline: Grant/Research Support|Moderna: Grant/Research Support|Pfizer: Grant/Research Support **Hung Fu Tseng, PhD MPH**, GSK: Grant/Research Support|Moderna: Grant/Research Support

